# Nanoscale Quantum Thermal Conductance at Water Interface: Green’s Function Approach Based on One-Dimensional Phonon Model

**DOI:** 10.3390/molecules25051185

**Published:** 2020-03-05

**Authors:** Toshihito Umegaki, Shigenori Tanaka

**Affiliations:** Graduate School of System Informatics, Kobe University, 1-1 Rokkodai-cho, Nada-ku, Kobe 657-8501, Japan

**Keywords:** quantum thermal conductance, water, nanoscale, Green’s function, phonon

## Abstract

We have derived the fundamental formula of phonon transport in water for the evaluation of quantum thermal conductance by using a one-dimensional phonon model based on the nonequilibrium Green’s function method. In our model, phonons are excited as quantum waves from the left or right reservoir and propagate from left to right of H${}_{2}$O layer or vice versa. We have assumed these reservoirs as being of periodic structures, whereas we can also model the H${}_{2}$O sandwiched between these reservoirs as having aperiodic structures of liquid containing *N* water molecules. We have extracted the dispersion curves from the experimental absorption spectra of the OH stretching and intermolecular modes of water molecules, and calculated phonon transmission function and quantum thermal conductance. In addition, we have simplified the formulation of the transmission function by employing a case of one water molecule (*N*=1). From this calculation, we have obtained the characteristic that the transmission probability is almost unity at the frequency bands of acoustic and optical modes, and the transmission probability vanishes by the phonon attenuation reflecting the quantum tunnel effect outside the bands of these two modes. The classical limit of the thermal conductance calculated by our formula agreed with the literature value (order of $10^{-10}$ W/K) in high temperature regime (>300 K). The present approach is powerful enough to be applicable to molecular systems containing proteins as well, and to evaluate their thermal conductive characteristics.

## 1. Introduction

In recent years, advances in the fabrication and characterization of nanoscale systems allow for a better understanding of the heat flow at the microscopic level [1]. Regarding the researches for inorganic materials, studies on carbon nanotube systems have provided a prototypical example for nanoscale thermal conduction experimentally [2,3,4,5,6] and theoretically [7,8]. Both theoretical and experimental progresses have thus been reported extensively in each of these studies [1]. As for other nanoscale systems, Lervik et al. [9] analyzed via classical molecular dynamics simulations the heat transfer through nanometer-scale interfaces consisting of n-decane (2–12 nm diameter) droplets in water. Tanaka et al. [10,11] studied molecular dynamics of water by microwave heating. In contrast to these classical-mechanics-based approaches, Fisher [12] showed that the Landauer transport formalism can be applied to the formulations of the quantum thermal conductance of heat flux carried by phonons between hot and cold reservoirs on the basis of nanoscale models of inorganic materials. Furthermore, using the Landauer formulation, Rego et al. [13] studied the quantized thermal conductance of dielectric quantum wires at low temperatures.

On the other hand, attention has recently been focused on the nanoscale thermal conduction in biological systems as well. Concerning experimental reports on endogenous heat production in single cells, Baffou et al. [14] argued that the experimentally observed temperature rise of ΔT (1–2 K) of a whole cell [15,16,17] would be much larger than the theoretical ΔT estimated from the relation ΔT=P/κL, where κ is the thermal conductivity, *L* is the diameter of the heat source and *P* is the power. This equation is derived from the macroscopic heat diffusion equation for continuum [18]. Baffou et al. [19] estimated ΔT within a cell to be around 10${}^{-5}$ K. However, the 10${}^{5}$ gap issue (the difference between the measured temperature increase of 1 K in a single cell using cellular thermometry as compared to the theoretically calculated increase estimate of 10${}^{-5}$ K) may disappear [15] if once the uncertainties of variable heat sources in stimulated cells (10${}^{1}$), length scales (10${}^{1}$–10${}^{2}$), and micro- and nanoscale thermal parameters (10${}^{1}$–10${}^{2}$) are considered. A forthcoming challenge in the series of debates [14,15] is thus to establish a relevant theoretical framework for thermal conduction by setting up nanoscale models of biological cells containing liquid water and proteins. Regarding this issue, Lervik et al. [20] calculated the thermal conductivity at the protein–water interface in terms of classical molecular dynamics. Pandey and Leitner [21,22] quantum-mechanically evaluated the thermal energy transport through a trehalose layer between water and protein, and between gold, such as a gold nanoparticle, and its cellular environment.

In this paper, we aim at constructing a theoretical formulation to describe the quantum thermal conduction at nanoscale, paying special attention to the roles by water in biological contexts. We here derive the fundamental formula of phonon transport in water for the first step of evaluations of quantum thermal conductances in intracellular molecular environment. In the nanoscale space relevant to intracellular environment, we expect that the quantum characteristics of heat transfer may manifest themselves, which should be compared to the classical descriptions. The model of this study, which is given by a novel combination of the Landauer formulation and spectroscopic data, is powerful enough to be applicable to molecular systems containing proteins, and to quantitatively evaluate the thermal conductive characteristics in realistic systems, since experimental spectroscopic features are taken into account in the model. In the present formulation, we have applied the Green’s function method (GFM) to water with a one-dimensional phonon model. This model then takes into account not only intramolecular vibration modes but also intermolecular ones. We have extracted the dispersion curves from experimental absorption spectrum of water and evaluated quantitatively quantum heat conduction characteristics such as the phonon transmission function and the thermal conductance. Based on the GFM theoretical framework and the extracted parameters, we have calculated the quantum thermal conductance and compared it with the literature values quantitatively. We show detailed mathematical formulations based on equilibrium Green’s functions, non-equilibrium Green’s functions and phonon transmission function in the Appendix A, and describe simplified formulas used in the calculations in the following sections of main text.

## 2. Extraction of Phonon Dispersion Curves of Liquid Water from Experimental Results

**Figure 1:**
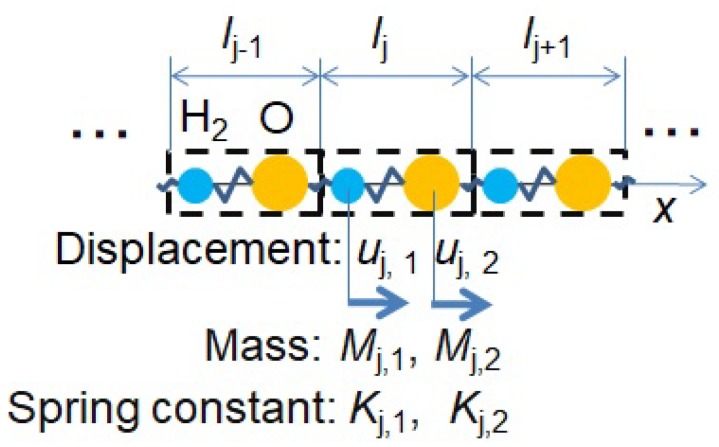
Definitions of displacements, masses, and spring constants of the elements H${}_{2}$ (blue) and O (yellow) in the one-dimensional phonon model.

**Figure 2:**
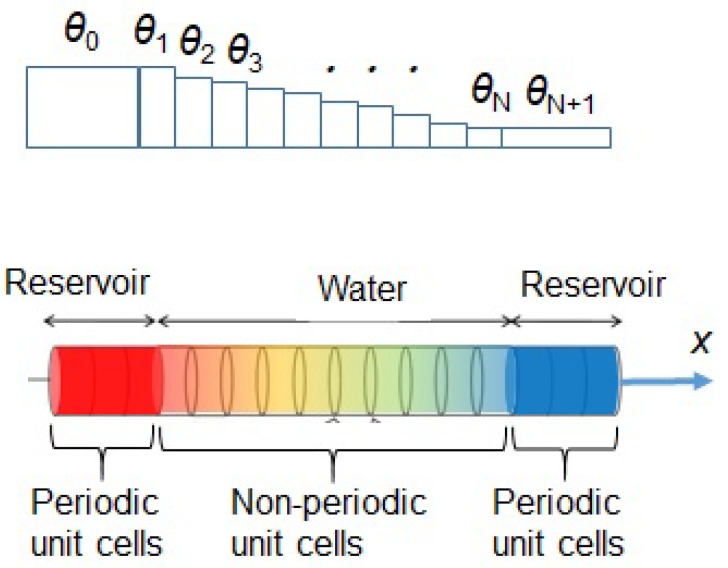
One-dimensional phonon model of reservoir-water-reservoir structure. Each unit cell *j* has length $l_{j}$, ($j=-N_{L}+1,-N_{L}+2,\cdots N+N_{R}$) along the x-direction and temperature $\theta_{j}$, (j=0,1,2,⋯N+1), where $\theta_{-N_{L}+1}=\theta_{-N_{L}+2}=\cdots =\theta_{0}$ and $\theta_{N+1}=\theta_{N+2}=\cdots =\theta_{N+N_{R}}$ [23].

Figure 1 illustrates the definitions of displacements, masses, and spring constants of the elements H${}_{2}$ and O in the present one-dimensional phonon model. Figure 1 gives a background of the theoretical formulations. The *j*-th unit cell of water molecule with length $l_{j}$ consists of two elements, H${}_{2}$ and O, blue and yellow colored circles, which have displacements $u_{j,1}$ and $u_{j,2}$, masses $M_{j,1}$ and $M_{j,2}$, and spring constants $K_{j,1}$ and $K_{j,2}$, respectively. By applying the Bloch theorem $u_{j+1,n}=\exp\left(ikl_{j}\right)u_{j,n}$ to Equations (A1a)–(A2b) in Appendix A, we can obtain a phonon dispersion relation of liquid water between the frequency ω and the dimensionless wavenumber:

(1) $$kl_{j}=\arccos\left[\frac{2M_{j,1}^{-1}M_{j,2}^{-1}K_{j,1}K_{j,2}-\left(M_{j,1}^{-1}+M_{j,2}^{-1}\right)\left(K_{j,1}+K_{j,2}\right)\omega^{2}+\omega^{4}}{2M_{j,1}^{-1}M_{j,2}^{-1}K_{j,1}K_{j,2}}\right],$$

where *j* and *n* in $K_{j,n}$ and $M_{j,n}$ are indices of unit cells and atomic components, respectively. In the model of Figure 2, *j* and *n* take values from ($-N_{L}+1$) to ($N+N_{R}$) and 1, 2, respectively, where $N_{L}$, $N_{R}$, and *N* represent the number of unit cells in the left and right reservoirs and the water, respectively, and n=1,2 mean two-hydrogen (H${}_{2}$) and one-oxygen (O), respectively. The phonon dispersion relation (1) has corresponding dispersion curves with real *k* branches of acoustic and optical modes. Hereafter, assuming that $M_{j,1}$ and $M_{j,2}$ do not depend on *j*, we have approximated them by $M_{1}$ and $M_{2}$, respectively, and similarly, $K_{j,1}$ and $K_{j,2}$ were also approximated by $K_{1}$ and $K_{2}$, respectively. If we assume the acoustic and optical modes have angular frequency bands of 0 $\leq \omega \leq \omega_{r}$ and $\omega_{q}\leq \omega \leq \omega_{p}$, respectively, we can obtain equations for $K_{1}$, $K_{2}$ and $\omega_{p}$ as follows:

(2a) $$\begin{array}{cc} K_{1} & =\frac{1}{2}\left\{\frac{M_{1}M_{2}\left(\omega_{q}^{2}+\omega_{r}^{2}\right)}{M_{1}+M_{2}}+\sqrt{{\left[\frac{M_{1}M_{2}\left(\omega_{q}^{2}+\omega_{r}^{2}\right)}{M_{1}+M_{2}}\right]}^{2}-M_{1}M_{2}\omega_{q}^{2}\omega_{r}^{2}}\right\}, \end{array}$$

(2b) $$\begin{array}{cc} K_{2} & =\frac{1}{2}\left\{\frac{M_{1}M_{2}\left(\omega_{q}^{2}+\omega_{r}^{2}\right)}{M_{1}+M_{2}}-\sqrt{{\left[\frac{M_{1}M_{2}\left(\omega_{q}^{2}+\omega_{r}^{2}\right)}{M_{1}+M_{2}}\right]}^{2}-M_{1}M_{2}\omega_{q}^{2}\omega_{r}^{2}}\right\}, \end{array}$$

(2c) $$\begin{array}{cc} \omega_{p} & =\sqrt{M_{1}^{-1}K_{1}+M_{1}^{-1}K_{2}+M_{2}^{-1}K_{1}+M_{2}^{-1}K_{2}}. \end{array}$$

**Figure 3:**
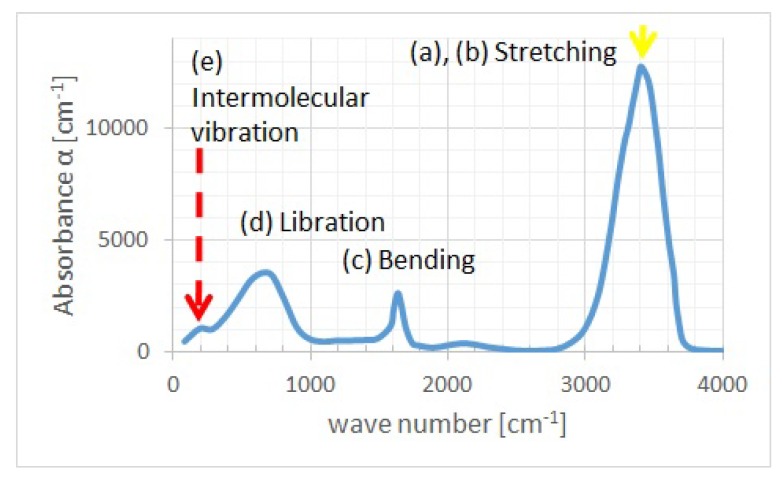
Absorption spectrum of water in the liquid phase [24]. Yellow and red broken lines represent the vibration modes (b) and (e), respectively.

**Figure 4:**
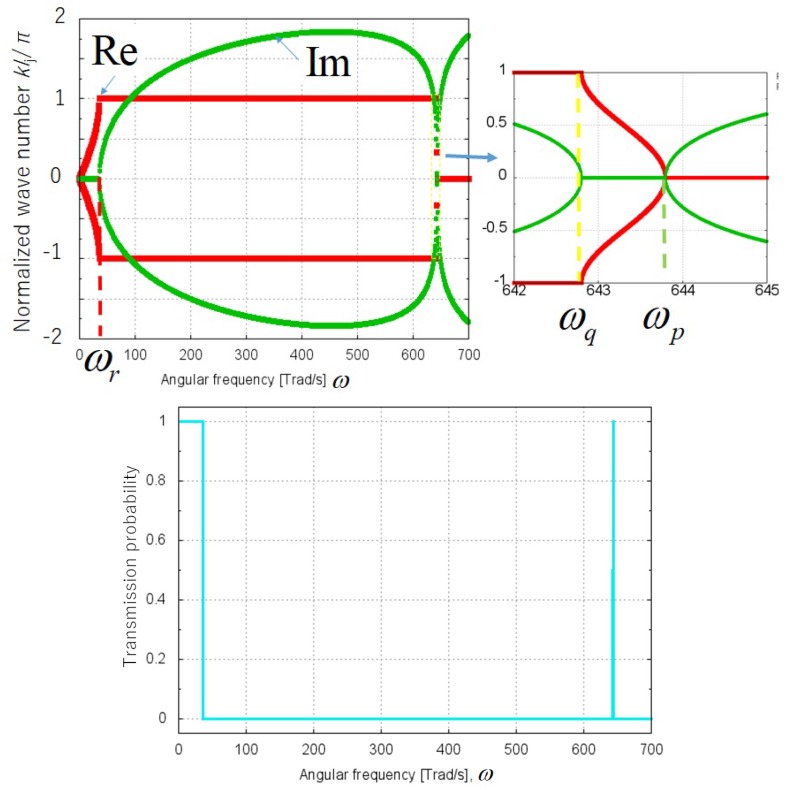
Complex dispersion curves (**upper**) and transmission probability spectrum of the phonon (**lower**). The upper right inset plots enlarged dispersion curves in the frequency range of 642–645 Trad/s.

As illustrated in Figure 2, we have assumed the model in which phonons are excited as quantum waves from the left or right reservoir and propagate from left to right of water layers or vice versa. These reservoirs have periodic structures of solid in the red and blue areas of Figure 2, whereas water may have an aperiodic structure of liquid in the rainbow colored area of Figure 2; the reason for employing the aperiodic structure is to generally describe disordered liquid water. We have assumed a one-dimensional phonon model with intervals of water molecule as $l_{j}$ in the *j*-th unit cell, where j=1,2,⋯N as shown in Figure 2. Each unit cell of water is modelled to consist of two components, H${}_{2}$ and O. The present model takes into account not only intramolecular vibration modes but also intermolecular ones. Phonon has two kinds of modes, acoustic and optical modes. The components of water molecules at acoustic and optical modes vibrate with the same and inverse directions, respectively. We have extracted the dispersion curves from the experimental absorption spectrum of H${}_{2}$O in the liquid phase [24] by the following procedure:

1. Extracting wave numbers $k_{b}$ and $k_{e}$ at yellow and red broken lines of absorbance maxima in the experimental absorption spectrum of water in the liquid phase shown in Figure 3.
2. Calculating angular frequencies $\omega_{q}$ and $\omega_{r}$ as $2\pi k_{b}c$ and $2\pi k_{e}c$, respectively, with *c* being the light velocity, so that the one-dimensional phonon model is consistent with the experimental spectrum.
3. Calculating spring constants $K_{1}$ and $K_{2}$ by using Equations (2a) and (2b) above.
4. Calculating angular frequency $\omega_{p}$ by using Equation (2c) above.
5. Calculating the wave number *k* of phonon by using the dispersion relation Equation (1) above.
6. Obtaining dispersion curves in the upper part of Figure 4 below with the frequency on the abscissa and the normalized wave number $kl_{j}/\pi$ on the ordinate.

**Figure 5:**
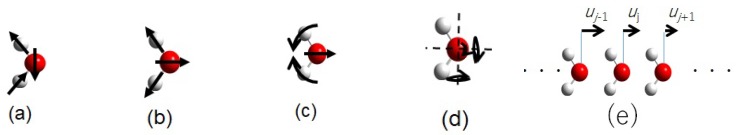
Modes of H${}_{2}$O molecular motions of (**a**) anti-symmetric stretching; (**b**) symmetric stretching; (**c**) bending; (**d**) libration; and (**e**) intermolecular vibration.

Figure 5 shows liquid-phase water motions [25,26] of (a) anti-symmetric stretching, (b) symmetric stretching, (c) bending, (d) libration, and (e) intermolecular vibration, respectively. In step 1, we determined wave numbers $k_{b}$ and $k_{e}$ as modes (b) and (e) from the maximum value of the experimental absorption spectrum shown in Figure 3, where we selected $k_{b}$ as maximum value of intramolecular vibration in the experimental spectrum because the absorbance of mode (b) was higher than that of mode (a) [27]. It is also noted that some other modes such as those associated with hydrogen bond stretch may be contained in the “libration” region (d) in Figure 3, while they are supposed to play an insignificant role in the present simplified model. This procedure is applicable not only to liquid water, but also to water in a wide range of solid, liquid, and gas temperatures [28]. Table 1 shows the wavenumbers and angular frequencies of various molecular vibrational modes of liquid water, where $k_{a}$, $k_{b}$, $k_{c}$, $k_{d}$, and $k_{e}$ are wavenumbers of liquid-phase water motions [25,26] of (a)–(e), respectively. Because of small difference between $k_{a}$ and $k_{b}$, we have calculated $k_{a}$ as $k_{a}^{\prime }-k_{b}^{\prime }+k_{b}$, where $k_{a}^{\prime }$ and $k_{b}^{\prime }$ are wave numbers of isolated water molecule 3756 cm${}^{-1}$ and 3657 cm${}^{-1}$, respectively [27].

## 3. Formulation of the Phonon Transmission Function through H${}_{2}$O

To obtain the physical outlook without losing physical essence, we have derived a simple expression for one H${}_{2}$O molecule on the basis of the formula derived from the equation of the phonon transmission function for a region consisting of *N* water molecules, described in Appendix A.

**Figure 6:**
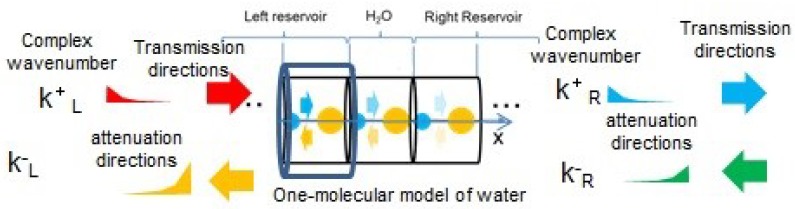
A model of phonon transport through a water molecule between two reservoirs.

In this section, we derive equations of the phonon transmission function tr(T) for H${}_{2}$ and O components of one water molecule, that is, the *N*=1 phonon model of Figure 6 for simplicity, and perform test calculations using the formula based on the *N*=1 water layer model without losing physical essence.

We set *N* = 1 in Equations (A12), (A14c), (A14d), (A15a), (A15b), (A23a), (A23b), (A24), and obtained the following equations:

(3a) $$\begin{array}{cc} tr(T) & =\Gamma_{L,11}\Gamma_{R,22}{\left|G_{D,12}\right|}^{2}, \end{array}$$

(3b) $$\begin{array}{cc} G_{D,12} & =\frac{-\gamma_{0,2}}{\begin{array}{c} \left(\varepsilon +i\eta -\varepsilon_{1,1}-\gamma_{1,1}\gamma_{0,4}g_{L,22}\right)\left(\varepsilon +i\eta -\varepsilon_{1,2}-\gamma_{N,4}\gamma_{N+1,1}g_{R,11}\right)-\gamma_{1,2}\gamma_{1,3} \end{array}}, \end{array}$$

(3c) ΓL,11=−2γ1,1γ0,4Im(gL,22),

(3d) ΓR,22=−2γN,4γN+1,1Im(gR,11),

(3e) $$\begin{array}{cc} g_{L,22} & =\frac{\varepsilon +i\eta -\varepsilon_{1,1}+\frac{\varepsilon_{1,1}-\varepsilon }{1-\exp\left(i\left(k_{L}^{+}-k_{L}^{-}\right)l_{0}\right)}}{\begin{array}{c} \left(\varepsilon +i\eta -\varepsilon_{0,1}+\frac{\varepsilon_{0,1}-\varepsilon }{1-\exp\left(i\left(k_{L}^{+}-k_{L}^{-}\right)l_{0}\right)}\right)\left(\varepsilon +i\eta -\varepsilon_{0,2}\right)-\left(\gamma_{0,2}-\frac{\gamma_{0,2}+\gamma_{0,1}\exp\left(-ik_{L}^{-}l_{0}\right)}{1-\exp\left(i\left(k_{L}^{+}-k_{L}^{-}\right)l_{0}\right)}\right)\gamma_{0,3} \end{array}}, \end{array}$$

(3f) $$\begin{array}{cc} g_{R,11} & =\frac{\varepsilon +i\eta -\varepsilon_{N,2}+\frac{\varepsilon_{N,2}-\varepsilon }{1-\exp\left(i\left(k_{R}^{+}-k_{R}^{-}\right)l_{N}\right)}}{\begin{array}{c} \left(\varepsilon +i\eta -\varepsilon_{N,2}+\frac{\varepsilon_{N,2}-\varepsilon }{1-\exp\left(i\left(k_{R}^{+}-k_{R}^{-}\right)l_{N}\right)}\right)\left(\varepsilon +i\eta -\varepsilon_{N,1}\right)-\left(\gamma_{N,3}-\frac{\gamma_{N+1,3}+\gamma_{N+1,4}\exp\left(ik_{R}^{+}l_{N}\right)}{1-\exp\left(i\left(k_{R}^{+}-k_{R}^{-}\right)l_{N}\right)}\right)\gamma_{N,2} \end{array}}, \end{array}$$

where tr means trace of matrix; $\Gamma_{L,jn}$, $\Gamma_{R,jn}$, $G_{D,jn}$, $g_{L,jn}$ and $g_{R,jn}$ (*j*, *n* = 1, 2) are jn elements of matrices $\Gamma_{L}$, $\Gamma_{R}$, $G_{D}$, $g_{L}$ and $g_{R}$, respectively; ε, $\varepsilon_{j,n}$ and $\gamma_{j,n}$ (*n* = 1–4) represent $\omega^{2}$, squared on-site energy and squared hopping energy, respectively, as defined in Equations (A2c)–(A2i) and (A6a)–(A6c) in Appendix A; Im(Z) is the imaginary part of complex number *Z*, and η is the imaginary part of the energy, which is a positive infinitesimal. T represents the transmission matrix:

(4) $$T=\Gamma_{L}G_{D}\Gamma_{R}G_{D}^{†},$$

as defined in Equation (A24) in Appendix A. The complex dispersion curves shown in the upper part of Figure 4 has three modes of (I) acoustic wave, (II) attenuation and (III) optical wave as shown in Table 2. In this study, we have employed an interval of water molecule $l_{1}$ as 0.319 nm [29] in Figure 2. We can calculate the transmission function by Equation (3a), and (I), (II) and (III) modes have the transmission probabilities of approximately 1, 0, 1 over angular frequency ranges of $0<\omega <\omega_{r}$, $\omega_{r}<\omega <\omega_{q}$, and $\omega_{q}<\omega <\omega_{p}$, respectively, as shown in Table 2. Then we can depict the frequency characteristics of the transmission probability as shown in the lower part of Figure 4. The transmission probability takes not only discrete values of 0 or 1, but continuous values between 0 and 1 near the boundary angular frequencies of $\omega_{r}$ and $\omega_{q}$.

## 4. Thermal Conductance in Water

### 4.1. Validation of Calculated Thermal Conductance

Since phonons are bosons, they accord to the Bose-Einstein distribution as follows:

(5) $$N_{j}\left(\omega \right)={\left\{\exp\left(\hbar \omega /k_{B}\theta_{j}\right)-1\right\}}^{-1},$$

where *ℏ*, ω, $k_{B}$, $\theta_{j}$ are reduced Planck constant, angular frequency, Boltzmann constant, and temperature in the *j*th unit cell of water molecules in the one-dimensional phonon model of Figure 2. We can thus formulate the Bose-Einstein distribution function at the unit cell *j* in one-dimensional phonon model of reservoir-H${}_{2}$O-reservoir structure shown in Figure 2. Moreover, we can define the heat flow *J* by the following equation [1,23,31]:

(6) $$J=\int_{0}^{\infty }d\omega \frac{\hbar \omega }{2\pi }\sum_{k=1}^{2}\sum_{j=1}^{N}{\left\{T\right\}}_{j,k;j,k}\left\{N_{0}\left(\omega \right)-N_{N+1}\left(\omega \right)\right\},$$

where the functions $N_{0}\left(\omega \right)$ and $N_{N+1}\left(\omega \right)$ are the Bose-Einstein distributions at left and right reservoirs, respectively. Then, thermal conductance σ is defined by an equation that divides the heat flow *J* by the temperature difference $\theta_{0}-\theta_{N+1}$ as follows:

(7) $$\sigma =\frac{J}{\theta_{0}-\theta_{N+1}}.$$

**Figure 7:**
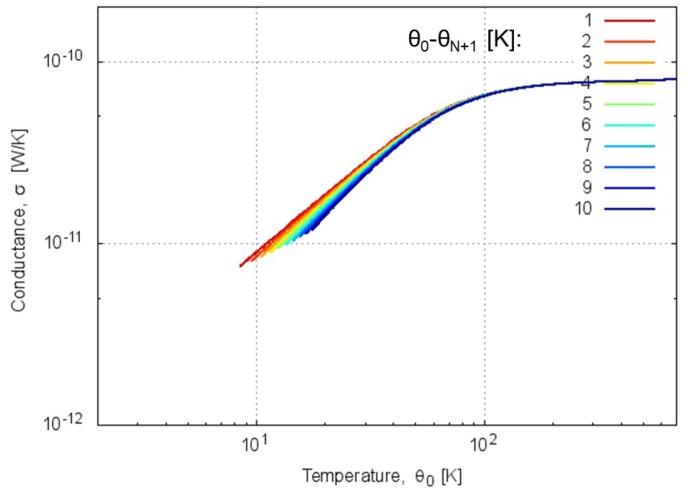
Temperature dependences of thermal conductance calculated by Equation (7). The parameters 1, 2, ⋯ 10 with unit of degrees Kelvin in the graph mean temperature differences $\theta_{0}-\theta_{N+1}$ between the left and right reservoirs.

Figure 7 shows temperature dependencies of thermal conductances by this calculation with Equation (7). The temperature dependences show fair agreement with the previous results [32] for homogeneous and heterogeneous atomic chains, where the mass of a ‘Device’ atom in the homogeneous case is 4.6 × 10${}^{-26}$ kg (Si atom assumed), and the masses of ‘Device’ atoms in the two heterogeneous cases are 9.2 and 2.3 × 10${}^{-26}$ kg, respectively. It is observed that the agreement is in particular better when the temperature difference $\left|\theta_{0}-\theta_{N+1}\right|$ is smaller. It is noted that the conductance curves in Figure 7 are not so sensitive to the atomic masses employed in the calculations.

### 4.2. Classical Limit of Thermal Conductance

Because the higher-order terms ${\left(\hbar \omega /k_{B}\theta_{j}\right)}^{2}/2!+{\left(\hbar \omega /k_{B}\theta_{j}\right)}^{3}/3!+\cdots$ can be neglected in the classical limit $\hbar \omega /k_{B}\theta_{j}\ll 1$, the Bose-Einstein distribution function $N_{j}\left(\omega \right)$ of Equation (5) takes the following limiting form:

(8) $$N_{j}\left(\omega \right)={\left\{1+\left(\hbar \omega /k_{B}\theta_{j}\right)/1!+{\left(\hbar \omega /k_{B}\theta_{j}\right)}^{2}/2!+\cdots -1\right\}}^{-1}≅\frac{k_{B}\theta_{j}}{\hbar \omega }.$$

For example, in a case of temperature $\theta_{j}$=300K and angular frequency range $\omega \leq \omega_{r}$, we have $\hbar \omega /k_{B}\theta_{j}\leq 0.912$. In this case we may use the approximation of Equation (8). $\left\{N_{0}\left(\omega \right)-N_{N+1}\left(\omega \right)\right\}$ of Equation (6) can then be approximated to $\left(k_{B}/\hbar \omega \right)\left(\theta_{0}-\theta_{N+1}\right)$. The phonon transmission probability $\sum_{k=1}^{2}\sum_{j=1}^{N}{\left\{T\right\}}_{j,k;j,k}$ can be approximated to unity in the bands of $0<\omega <\omega_{r}$ and $\omega_{q}<\omega <\omega_{p}$, and zero for the other bands of ω according to the lower part of Figure 4. From these two approximations, the integral with respect to angular frequency of Equation (6) can be approximated by the following equation,

(9) $$\begin{array}{c} J=\frac{k_{B}}{2\pi }\left(\omega_{r}-\omega_{q}+\omega_{p}\right)\left(\theta_{0}-\theta_{N+1}\right)≅\frac{k_{B}\omega_{r}}{2\pi }\left(\theta_{0}-\theta_{N+1}\right), \end{array}$$

where we have transformed the 3rd expression in Equation (9) by using $\left|-\omega_{q}+\omega_{p}\right|\ll \omega_{r}$. Equation (9) can be used for the quantum heat flux due to phonon propagation at the molecular size in the cell, which may be formulated according to the actual size of cell biology. In the classical limit, thermal conductance σ is thus expressed by Equations (7) and (9) as follows:

(10) $$\sigma =\frac{k_{B}\omega_{r}}{2\pi }.$$

By using Equation (10), we can evaluate the thermal conductance in the classical limit as $\sigma =7.87\times 10^{-11}$ W/K, which agrees well with the values in the high-temperature limit in Figure 7 showing the order of 10${}^{-10}$ W/K; this evaluation is also similar to the estimated value of about 100 MW/K/m${}^{2}$ by Lervik et al. [20] with inclusion of the effective cross section (1 nm)${}^{2}$. The quantum thermal conductance evaluated in the present study thus agrees with the thermal conductance obtained via classical molecular dynamics simulation in the high-temperature, classical limit. One of significant results in the present study is that the magnitude of thermal conductance σ in the classical limit calculated from $\omega_{r}$ is similar to that by classical molecular dynamics for protein–water system, whereas the former would give somewhat lower estimate due to the neglect of the contributions from other modes.

Baffou et al. argued [19] that the observed temperature rise of ΔT (1-2 K) of a whole cell [16,17] would be much larger than the theoretical ΔT estimated from the relation ΔT=P/κL, where *L* is the diameter of the heat source, *P* is power, and κ is thermal conductivity estimated to be around 1 W/K/m [19]. They estimated ΔT within a cell to be around 10${}^{-5}$ K, assuming that the heat source size is 10 nm and that the average heat production per hour based on glucose production is 100 pW (360 nJ/h). However, one may expect that once three uncertainties below are supposed, the 10${}^{5}$ gap issue may disappear [15]:

1. variable heat sources in stimulated cells (order 10${}^{1}$);
2. length scales (order 10${}^{1}$–10${}^{2}$);
3. micro- and nanoscale thermal parameters (order 10${}^{1}$–10${}^{2}$).

Corresponding to these issues, we can make use of analytical model dicussed above. In order to assess these issues, in this paper, we constructed the following models in the framework of nonequilibrium Green’s function method:

1. reservoir model representing heat and bath reservoirs;
2. one-dimensional phonon model with water molecules sandwiched between the reservoirs;
3. phonon transports at atomic scale in water.

Thus Equation (7) can provide quantitative suggestions for the discussions on intracellular temperature distributions [14,15,19,33]. At physiological temperature, our quantum-mechanical estimation for the thermal conductance above is consistent with the values evaluated through classical molecular dynamics simulations for protein–water systems [20]. Then, if we would employ the evaluations of thermal conductivity κ by Lervik et al. [20] as well, the values of κ would fall around 0.1–0.3 W/K/m, which could make the discussions [14,15,19,33] more quantitative in nanoscale intracellular conditions. Of course, further theoretical investigations would be required to resolve the gap issue.

## 5. Conclusions

We have derived the fundamental formula of phonon transport in water for the first step of evaluations of quantum thermal conductance by using a one-dimensional phonon model. We have calculated the dispersion curves reproducing OH stretching mode of water molecule and inter-molecular mode, and reproduced experimental values of the absorption spectra by fitting the parameters of the phonon modes such as $\omega_{r}$, $\omega_{q}$ and so on. We also have formulated the phonon transmission function of *N* water molecules connected to the left and right heat baths by using the non-equilibrium Green’s function method. We have employed a model in which phonons are excited as quantum waves from the left or right reservoir and propagate from left to right of H${}_{2}$O layer or vice versa. We have modelled these reservoirs as having periodic structures, whereas we can regard the water layer part as aperiodic structure of liquid. In this formulation, we have applied the Green’s function method to H${}_{2}$O with a one-dimensional phonon model. The model of this formulation is powerful enough to be applicable to molecular system containing proteins, and to evaluate thermal conductive characteristics thereof.

We have extracted dispersion curves from experimental absorption spectrum of H${}_{2}$O and quantitatively evaluated quantum heat conduction characteristics such as the phonon transmission function and the thermal conductance. In addition, we have simplified the formulation of the transmission function by employing one water molecule (*N*=1) in the aperiodic layer. From this calculation, we have obtained the characteristic that the transmission probability is almost unity in the frequency bands of acoustic and optical modes, whereas the transmission probability vanishes reflecting the quantum tunnel effect in the frequency band between these two modes. The classical limit of the thermal conductance calculated by our formula and the literature value [32] in high temperature regime (>300 K) have agreed with each other on the order of $10^{-10}$W/K. The present model has also provided the calculated results for thermal conductance that are consistent with those evaluated via classical molecular dynamics simulations for protein–water systems [20] in the classical limit.

As future issues, in order to extract more information from the experimental results of water absorption spectra [34], we will expand our one-dimensional phonon model to higher-dimensional models including other vibration modes [35]. We expect this refinement of our model to provide more accurate reproduction of heat conduction characteristics. We are aiming at multi-scale heat conduction analysis by modeling not only water but also intracellular molecules such as proteins. We would like to apply the present method also to the analysis of heat conduction characteristics of biomolecules containing surrounding molecules such as flexible sugar chains [36], which would give more insights into the thermal conductive phenomena in intracellular crowding environments. 

## Figures and Tables

**Table 1:** Wavenumbers and angular frequencies for various molecular vibration modes of water (*c*: light velocity).

		Wave Number	Angular Frequency
Mode	α	$k_{\alpha }$ (cm${}^{-1}$)	2 π$k_{\alpha }c$ (Trad/s)
OH anti-symmetric	a	3509	661.4
stretching			
OH symmetric	b	3410	642.8
stretching			
OH bending	c	1660	312.9
Binding rotation	d	700	131.9
(libration)			
Intermolecular	e	190	35.8
vibration			

**Table 2:** Each vibrational mode (left), angular frequency range (center) and transmission probability (right) ${}^{†}$.

	Mode	Angular Frequency	Transmission
		Range	Probability
(I)	acoustic wave	$0<\omega <\omega_{r}$	≅1
(II)	attenuation	$\omega_{r}<\omega <\omega_{q}$	≅0
(III)	optical wave	$\omega_{q}<\omega <\omega_{p}$	≅1

†: (transmission probability) = tr(T)/(its maximum value over ω) [30].

## Abbreviations

The following abbreviations are used in this manuscript:

GFM	Green’s function method
Trad/s	unit of tera radians per second
tr	trace of matrix
Im	imaginary part of complex number

## Appendix A. Phonon Transport Equations in a One-Dimensional Chain Model of Water

We perform derivations in Appendix A.1: transport equations of phonon in water, Appendix A.2: dispersion equations in left and right reservoirs, Appendix A.3: non-equivribrium Green’s functions, and Appendix A.4: quantum thermal conductance in water in the following.

### Appendix A.1. Derivation of Transport Equations of Phonon in Water

We can formulate phonon transport equations in a one-dimensional model of water in terms of atomic displacements $u_{j,n}\left(t\right),j=-N_{L}+1,\cdots -1,0,1,\cdots N+N_{R}$ based on Figure 1 as follows:

(A1a) $$\begin{array}{cc} M_{j,1}\frac{d^{2}u_{j,1}\left(t\right)}{dt^{2}}= & -K_{j-1,2}\left[u_{j,1}\left(t\right)-u_{j-1,2}\left(t\right)\right]-K_{j,1}\left[u_{j,1}\left(t\right)-u_{j,2}\left(t\right)\right], \end{array}$$

(A1b) $$\begin{array}{cc} M_{j,2}\frac{d^{2}u_{j,2}\left(t\right)}{dt^{2}}= & -K_{j,1}\left[u_{j,2}\left(t\right)-u_{j,1}\left(t\right)\right]-K_{j,2}\left[u_{j,2}\left(t\right)-u_{j+1,1}\left(t\right)\right], \end{array}$$

where n=1,2 in $u_{j,n}\left(t\right)$ mean two-hydrogen (H${}_{2}$) and one-oxygen (O) atomic components, respectively. This model can mimic not only intramolecular vibration modes but also intermolecular ones. We can obtain time-independent equations of Fourier transforms of Equations (A1a) and (A1b) as follows.

(A2a) $$\begin{array}{cc} \; & -\gamma_{j,1}{\hat{u}}_{j-1,2}\left(\omega \right)+\varepsilon_{j,1}{\hat{u}}_{j,1}-\gamma_{j,2}{\hat{u}}_{j,2}\left(\omega \right)=\varepsilon {\hat{u}}_{j,1}\left(\omega \right), \end{array}$$

(A2b) $$\begin{array}{cc} \; & -\gamma_{j,3}{\hat{u}}_{j,1}\left(\omega \right)+\varepsilon_{j,2}{\hat{u}}_{j,2}-\gamma_{j,4}{\hat{u}}_{j+1,1}\left(\omega \right)=\varepsilon {\hat{u}}_{j,2}\left(\omega \right), \end{array}$$

(A2c) $$\begin{array}{cc} \; & \varepsilon =\omega^{2}, \end{array}$$

(A2d) $$\begin{array}{cc} \; & \varepsilon_{j,1}=\left(K_{j-1,2}+K_{j,1}\right)/M_{j,1}, \end{array}$$

(A2e) $$\begin{array}{cc} \; & \varepsilon_{j,2}=\left(K_{j,1}+K_{j,2}\right)/M_{j,2}, \end{array}$$

(A2f) $$\begin{array}{cc} \; & \gamma_{j,1}=K_{j-1,2}/M_{j,1}, \end{array}$$

(A2g) $$\begin{array}{cc} \; & \gamma_{j,2}=K_{j,1}/M_{j,1}, \end{array}$$

(A2h) $$\begin{array}{cc} \; & \gamma_{j,3}=K_{j,1}/M_{j,2}, \end{array}$$

(A2i) $$\begin{array}{cc} \; & \gamma_{j,4}=K_{j,2}/M_{j,2}, \end{array}$$

where ${\hat{u}}_{j,n}\left(\omega \right)$ is the Fourier transform of $u_{j,n}$(*t*) formulated as

(A3) $${\hat{u}}_{j,n}\left(\omega \right)=\frac{1}{\sqrt{2\pi }}\int dtu_{j,n}\left(t\right)\exp\left(i\omega t\right),$$

$\varepsilon_{j,1}$ and $\varepsilon_{j,2}$ represent squared on-site energy, and $\gamma_{j,1}$, $\gamma_{j,2}$, $\gamma_{j,3}$ and $\gamma_{j,4}$ represent squared hopping energy, respectively. By using Equations (A2a) and (A2b) with the left reservoir $j=-N_{L}+1,-N_{L}+2,\cdots -1,0$, the H${}_{2}$O array j=1,2,⋯N, and the right reservoir $j=N+1,\cdots N+N_{R}$ of whole structure shown in Figure 2, we can obtain phonon transport equations in matrix forms as follows:

(A4a) $$\begin{array}{cc} \; & \left(\begin{array}{ccc} H_{L} & \tau_{LD} & 0\\ \tau_{DL} & H_{D} & \tau_{DR}\\ 0 & \tau_{RD} & H_{R} \end{array}\right)\left(\begin{array}{c} {\hat{u}}_{L}\\ {\hat{u}}_{D}\\ {\hat{u}}_{R} \end{array}\right)=\varepsilon \left(\begin{array}{c} {\hat{u}}_{L}\\ {\hat{u}}_{D}\\ {\hat{u}}_{R} \end{array}\right), \end{array}$$

(A4b) $$\begin{array}{cc} \; & H_{L}=\left(\begin{array}{cccc} H_{-N_{L}+1} & V_{-N_{L}+1} & \; & 0\\ W_{-N_{L}+2} & H_{-N_{L}+2} & \ddots \\ \; & \ddots & \ddots & V_{-1}\\ 0 & \; & W_{0} & H_{0} \end{array}\right), \end{array}$$

(A4c) $$\begin{array}{cc} \; & H_{D}=\left(\begin{array}{cccc} H_{1} & V_{1} & \; & 0\\ W_{2} & H_{2} & \ddots \\ \; & \ddots & \ddots & V_{N-1}\\ 0 & \; & W_{N} & H_{N} \end{array}\right), \end{array}$$

(A4d) $$\begin{array}{cc} \; & H_{R}=\left(\begin{array}{cccc} H_{N+1} & V_{N+1} & \; & 0\\ \ddots & \ddots & \ddots \\ \; & W_{N+N_{R}-1} & H_{N+N_{R}-1} & V_{N+N_{R}-1}\\ 0 & \; & W_{N+N_{R}} & H_{N+N_{R}} \end{array}\right), \end{array}$$

(A4e) $$\begin{array}{cc} \; & \tau_{LD}=\left(\begin{array}{cc} \; & 0\\ V_{0} \end{array}\right), \end{array}$$

(A4f) $$\begin{array}{cc} \; & \tau_{DR}=\left(\begin{array}{cc} \; & 0\\ V_{N} \end{array}\right), \end{array}$$

(A4g) $$\begin{array}{cc} \; & \tau_{DL}=\left(\begin{array}{cc} \; & W_{1}\\ 0 \end{array}\right), \end{array}$$

(A4h) $$\begin{array}{cc} \; & \tau_{RD}=\left(\begin{array}{cc} \; & W_{N+1}\\ 0 \end{array}\right). \end{array}$$

Atomic displacement vectors $u_{L}$, $u_{D}$, and $u_{R}$ consist of $u_{j}$ of the *j*-th unit cell of each water molecule in the left reservoir, the H${}_{2}$O array, and the right reservoir, respectively.

(A5a) $$\begin{array}{cc} \; & {\hat{u}}_{L}=\left(\begin{array}{c} {\hat{u}}_{-N_{L}+1}\\ \vdots \\ {\hat{u}}_{-1}\\ {\hat{u}}_{0} \end{array}\right), \end{array}$$

(A5b) $$\begin{array}{cc} \; & {\hat{u}}_{D}=\left(\begin{array}{c} {\hat{u}}_{1}\\ {\hat{u}}_{2}\\ \vdots \\ {\hat{u}}_{N} \end{array}\right), \end{array}$$

(A5c) $$\begin{array}{cc} \; & {\hat{u}}_{R}=\left(\begin{array}{c} {\hat{u}}_{N+1}\\ {\hat{u}}_{N+2}\\ \vdots \\ {\hat{u}}_{N+N_{R}} \end{array}\right), \end{array}$$

(A5d) $$\begin{array}{cc} \; & {\hat{u}}_{j}=\left(\begin{array}{c} {\hat{u}}_{j,1}\\ {\hat{u}}_{j,2} \end{array}\right). \end{array}$$

The 2 × 2 matrices above consist of on-site energy and hopping energy of atoms in each water molecule of the *j*-th unit cell which are defined by following equations:

(A6a) $$\begin{array}{cc} \; & H_{j}=\left(\begin{array}{cc} \varepsilon_{j,1} & -\gamma_{j,2}\\ -\gamma_{j,3} & \varepsilon_{j,2} \end{array}\right), \end{array}$$

(A6b) $$\begin{array}{cc} \; & V_{j}=\left(\begin{array}{cc} 0 & 0\\ -\gamma_{j,4} & 0 \end{array}\right), \end{array}$$

(A6c) $$\begin{array}{cc} \; & W_{j}=\left(\begin{array}{cc} 0 & -\gamma_{j,1}\\ 0 & 0 \end{array}\right). \end{array}$$

### Appendix A.2. Derivation of Eigenvalue Equations in Left and Right Reservoirs

We define κ and λ as Fourier transforms of atomic displacements of ±x propagation waves in the left reservoir, and μ and ν as those in the right reservoir, respectively. We assume that the Fourier transforms of atomic displacements ${\hat{u}}_{j,n}$ are linear combinations of ±x propagating waves in the left and right reservoirs as follows:

(A7a) $$\begin{array}{c} {\hat{u}}_{j,n}=\left\{\begin{array}{cc} T_{j}\kappa_{n}+R_{j}\lambda_{n}, & -N_{L}+1\leq j\leq 0,n=1,2\\ T_{j}\mu_{n}+R_{j}\nu_{n}, & N+1\leq j\leq N+N_{R},n=1,2 \end{array}\right. \end{array}$$

where $T_{j}$ and $R_{j}$ ($-N_{L}+1\leq j\leq 0$ and $N+1\leq j\leq N+N_{R}$) are amplitudes of + and -x propagating waves, respectively.

Assuming that the phonon wavenumbers in the left and right reservoirs are $k_{L}^{\pm }$ and $k_{R}^{\pm }$, respectively, we derive the following equations from the periodic boundary conditions of amplitude based on Bloch’s theorem for the left and right reservoirs:

(A8a) $$\begin{array}{cc} T_{-1} & =\exp\left(-ik_{L}^{+}l_{0}\right)T_{0}, \end{array}$$

(A8b) $$\begin{array}{cc} T_{1} & =\exp\left(ik_{L}^{+}l_{0}\right)T_{0}, \end{array}$$

(A8c) $$\begin{array}{cc} R_{-1} & =\exp\left(-ik_{L}^{-}l_{0}\right)R_{0}, \end{array}$$

(A8d) $$\begin{array}{cc} R_{1} & =\exp\left(ik_{L}^{-}l_{0}\right)R_{0}, \end{array}$$

(A8e) $$\begin{array}{cc} T_{N+2} & =\exp\left(ik_{R}^{+}l_{N+1}\right)T_{N+1}, \end{array}$$

(A8f) $$\begin{array}{cc} T_{N} & =\exp\left(-ik_{R}^{+}l_{N+1}\right)T_{N+1}, \end{array}$$

(A8g) $$\begin{array}{cc} R_{N+2} & =\exp\left(+ik_{R}^{-}l_{N+1}\right)R_{N+1}, \end{array}$$

(A8h) $$\begin{array}{cc} R_{N} & =\exp\left(-ik_{R}^{-}l_{N+1}\right)R_{N+1}. \end{array}$$

In the left reservoir, applying $R_{j}$ = 0 and $T_{j}$ = 0 as the condition of no phonon propagating to ∓x directions, we obtain eigenvalue equations of plane waves of phonon propagating to ±x directions.

(A9a) $$\begin{array}{ccc} \; & \; & \left(\begin{array}{cc} \varepsilon_{0,1}-\varepsilon & -\gamma_{0,2}-\gamma_{0,1}\exp\left(-ik_{L}^{+}l_{0}\right)\\ -\gamma_{0,3}-\gamma_{0,4}\exp\left(ik_{L}^{+}l_{0}\right) & \varepsilon_{0,2}-\varepsilon \end{array}\right)\left(\begin{array}{c} \kappa_{1}\\ \kappa_{2} \end{array}\right)=\left(\begin{array}{c} 0\\ 0 \end{array}\right), \end{array}$$

(A9b) $$\begin{array}{ccc} \; & \; & \left(\begin{array}{cc} \varepsilon_{0,1}-\varepsilon & -\gamma_{0,2}-\gamma_{0,1}\exp\left(-ik_{L}^{-}l_{0}\right)\\ -\gamma_{0,3}-\gamma_{0,4}\exp\left(ik_{L}^{-}l_{0}\right) & \varepsilon_{0,2}-\varepsilon \end{array}\right)\left(\begin{array}{c} \lambda_{1}\\ \lambda_{2} \end{array}\right)=\left(\begin{array}{c} 0\\ 0 \end{array}\right). \end{array}$$

Similarly, in the right reservoir, applying $R_{j}$ = 0 and $T_{j}$ = 0 as the condition of no phonon propagating to ∓x direction, we obtain eigenvalue equations of plane waves of phonon propagating to ±x directions.

(A10a) $$\begin{array}{c} \left(\begin{array}{cc} \varepsilon_{N+1,1}-\varepsilon & -\gamma_{R1}^{+}\\ -\gamma_{R2}^{+} & \varepsilon_{N,2}-\varepsilon \end{array}\right)\left(\begin{array}{c} \mu_{1}\\ \mu_{2} \end{array}\right)=\left(\begin{array}{c} 0\\ 0 \end{array}\right), \end{array}$$

(A10b) $$\begin{array}{c} \left(\begin{array}{cc} \varepsilon_{N+1,1}-\varepsilon & -\gamma_{R1}^{-}\\ -\gamma_{R2}^{-} & \varepsilon_{N,2}-\varepsilon \end{array}\right)\left(\begin{array}{c} \nu_{1}\\ \nu_{2} \end{array}\right)=\left(\begin{array}{c} 0\\ 0 \end{array}\right), \end{array}$$

(A10c) $$\begin{array}{c} \gamma_{R1}^{\pm }\equiv \gamma_{N+1,2}+\gamma_{N+1,1}\exp\left(-ik_{R}^{\pm }l_{N+1}\right), \end{array}$$

(A10d) $$\begin{array}{c} \gamma_{R2}^{\pm }\equiv \gamma_{N+1,3}+\gamma_{N+1,4}\exp\left(ik_{R}^{\pm }l_{N+1}\right). \end{array}$$

We can calculate dispersion curves as solutions to the eigenvalue Equations (A9a)–(A10b) in the left and right reservoirs, respectively, and these dispersion curves are the same as the solutions of the dispersion relation (1).

### Appendix A.3. Non-Equilibrium Green’s Functions

The purpose of this section is to formulate phonon waves propagating in the water excited from left and right reservoirs, where we use the non-equilibrium Green’s functions defined by arranging Equation (A4a) [23]:

(A11) $$\begin{array}{c} \left(\begin{array}{ccc} G_{L} & G_{LD} & 0\\ G_{DL} & G_{D} & G_{DR}\\ 0 & G_{RD} & G_{R} \end{array}\right)={\left(\begin{array}{ccc} \left(\varepsilon +i\eta \right)I-H_{L} & -\tau_{LD} & 0\\ -\tau_{DL} & \left(\varepsilon +i\eta \right)I-H_{D} & -\tau_{DR}\\ 0 & -\tau_{RD} & \left(\varepsilon +i\eta \right)I-H_{R} \end{array}\right)}^{-1}, \end{array}$$

where $G_{L}$, $G_{D}$, and $G_{R}$ are retarded Green’s functions in the left reservoir, water molecules, and right reservoir, respectively; $G_{LD}$, $G_{DL}$, $G_{RD}$, and $G_{DR}$ are retarded Green’s functions at the interfaces between each reservoir and water. η is the imaginary part of the energy, which is a positive infinitesimal. Eliminating $G_{L}$, $G_{R}$, $G_{LD}$, $G_{DL}$, $G_{RD}$, and $G_{DR}$ from Equation (A11), we can get an expression of $G_{D}$ as follows:

(A12) $$G_{D}={\left[\left(\varepsilon +i\eta \right)I-H_{D}-\Sigma_{L}-\Sigma_{R}\right]}^{-1},$$

where $\Sigma_{L}$ and $\Sigma_{R}$ are self-energies defined as

(A13a) $$\begin{array}{cc} \Sigma_{L} & =\tau_{DL}g_{L}\tau_{LD}, \end{array}$$

(A13b) $$\begin{array}{cc} \Sigma_{R} & =\tau_{DR}g_{R}\tau_{RD}. \end{array}$$

$g_{L}$ and $g_{R}$ are equilibrium Green’s functions defined as follows:

(A14a) $$\begin{array}{cc} \; & g_{L}={\left[\left(\varepsilon +i\eta \right)I-H_{L}\right]}^{-1}, \end{array}$$

(A14b) $$\begin{array}{cc} \; & g_{R}={\left[\left(\varepsilon +i\eta \right)I-H_{R}\right]}^{-1}, \end{array}$$

(A14c) $$\begin{array}{cc} \; & g_{L}=\left(\begin{array}{cccc} g_{-N_{L}+1,-N_{L}+1} & g_{-N_{L}+1,-N_{L}+2} & \cdots & g_{-N_{L}+1,0}\\ g_{-N_{L}+2,-N_{L}+1} & g_{-N_{L}+2,-N_{L}+2} & \cdots & g_{-N_{L}+2,0}\\ \vdots & \; & \ddots & \vdots \\ g_{0,-N_{L}+1} & g_{0,-N_{L}+2} & \cdots & g_{0,0} \end{array}\right), \end{array}$$

(A14d) $$\begin{array}{cc} \; & g_{R}=\left(\begin{array}{cccc} g_{N+1,N+1} & g_{N+1,N+2} & \cdots & g_{N+1,N+N_{R}}\\ g_{N+2,N+1} & g_{N+2,N+2} & \cdots & g_{N+2,N+N_{R}}\\ \vdots & \; & \ddots & \vdots \\ g_{N+N_{R},N+1} & g_{N+N_{R},N+2} & \cdots & g_{N+N_{R},N+N_{R}} \end{array}\right), \end{array}$$

(A14e) $$\begin{array}{cc} \; & g_{j,j^{\prime }}=\left(\begin{array}{cc} g_{j,1;j^{\prime },1} & g_{j,1;j^{\prime },2}\\ g_{j,2;j^{\prime },1} & g_{j,2;j^{\prime },2} \end{array}\right),\;\;j,j^{\prime }=-N_{L}+1,-N_{L}+2,\cdots ,0,N+1,N+2,\cdots ,N+N_{R}. \end{array}$$

Substituting Equations (A4e)–(A4h), (A14c) and (A14d) into (A13a) and (A13b), we can obtain the following equations for the self-energies:

(A15a) $$\begin{array}{cc} \Sigma_{L} & =\left(\begin{array}{cc} W_{0}g_{0,0}V_{0} & 0\\ 0 & 0 \end{array}\right), \end{array}$$

(A15b) $$\begin{array}{cc} \Sigma_{R} & =\left(\begin{array}{cc} 0 & 0\\ 0 & V_{N}g_{N+1,N+1}W_{N+1} \end{array}\right). \end{array}$$

We can define the equilibrium Green’s function equivalent for each unit cell at each atomic position (*n*, $n^{\prime }$) in the left and right heat reservoirs as follows:

(A16a) $$\begin{array}{cc} g_{n,n^{\prime }}^{L} & =g_{j,n;j^{\prime },n^{\prime }},\;\;j,j^{\prime }=-N_{L}+1,-N_{L}+2,\cdots ,0, \end{array}$$

(A16b) $$\begin{array}{cc} g_{n,n^{\prime }}^{R} & =g_{j,n;j^{\prime },n^{\prime }},\;\;j,j^{\prime }=N+1,N+2,\cdots ,N+N_{R}. \end{array}$$

By executing the multiplications (A6c)${}_{j=1}$$g_{0,0}$ (A6b)${}_{j=0}$ and (A6b)${}_{j=N}$$g_{N+1,N+1}$ (A6c)${}_{j=N+1}$, non-zero components in the right hand side of (A15a) and (A15b) become

(A17a) $$\begin{array}{cc} W_{1}g_{0,0}V_{0} & =\left(\begin{array}{cc} \gamma_{1,1}g_{2,2}^{L}\gamma_{0,4} & 0\\ 0 & 0 \end{array}\right), \end{array}$$

(A17b) $$\begin{array}{cc} V_{N}g_{N+1,N+1}W_{N+1} & =\left(\begin{array}{cc} 0 & 0\\ 0 & \gamma_{N,4}g_{1,1}^{R}\gamma_{N+1,1} \end{array}\right). \end{array}$$

### Appendix A.4. Quantum Thermal Conductance in Water

Let us formulate equilibrium Green’s functions in reservoir, $g_{L}$, $g_{R}$, by the the following procedure consisting of basis transformations and so on:

1. Transforming wave functions consisting of the tight binding (TB) basis into those consisting of the plane wave (PW) basis.
2. Finding the PW basis solutions in semi-infinite ($N_{L}$, $N_{R}$→*∞*) heat reservoir at both ends of water with using the periodic characteristics of the heat reservoir in thermal equilibrium.
3. Inverse transformation from PW basis wave functions to TB basis ones.

Based on the components of the *j*=1st and *N*th unit cells of Equation (A4a), we can obtain the following equations governing the plane waves propagating to *x* direction in the left and right heat reservoirs, respectively:

(A18a) $$\begin{array}{cc} \varepsilon u_{1} & =W_{1}U_{L}P_{L}U_{L}^{-1}u_{1}+H_{1}u_{1}+V_{1}u_{0}, \end{array}$$

(A18b) $$\begin{array}{cc} \varepsilon u_{N} & =W_{N}u_{N-1}+H_{N}u_{N}+V_{N}U_{R}P_{R}U_{R}^{-1}u_{N}. \end{array}$$

We define $P_{L}$ and $P_{R}$ in Equations (A18a) and (A18b) as matrices in which (1,1) and (2,2) components of the diagonal terms are lined with propagation constants along *x* direction in the left and right heat reservoirs, respectively:

(A19a) $$\begin{array}{cc} P_{L} & =\left(\begin{array}{cc} \exp\left(-ik_{L}^{+}l_{0}\right) & 0\\ 0 & 0 \end{array}\right), \end{array}$$

(A19b) $$\begin{array}{cc} P_{R} & =\left(\begin{array}{cc} 0 & 0\\ 0 & \exp\left(ik_{R}^{-}l_{N+1}\right) \end{array}\right). \end{array}$$

Next, we define the matrices $U_{L}$ and $U_{R}$ in Equations (A18a) and (A18b) as unitary matrices consisting of the eigenvectors of the eigenvalue Equations (A9a)– (A10b) as follows:

(A20a) $$\begin{array}{cc} U_{L} & =\left(\begin{array}{cc} \kappa_{1} & \lambda_{1}\\ \kappa_{2} & \lambda_{2} \end{array}\right), \end{array}$$

(A20b) $$\begin{array}{cc} U_{R} & =\left(\begin{array}{cc} \mu_{1} & \nu_{1}\\ \mu_{2} & \nu_{2} \end{array}\right). \end{array}$$

We can simplify $g_{L}$ and $g_{R}$ as matrices with the same number of components as the number of the lattice points connected to the left and right heat reservoirs by using Equations (A18a) and (A18b), respectively:

(A21a) $$\begin{array}{cc} g_{L} & =g_{0,0}={\left[\left(\varepsilon +i\eta \right)I-H_{1}-W_{1}U_{L}P_{L}U_{L}^{-1}\right]}^{-1}, \end{array}$$

(A21b) $$\begin{array}{cc} g_{R} & =g_{N+1,N+1}={\left[\left(\varepsilon +i\eta \right)I-H_{N}-V_{N}U_{R}P_{R}U_{R}^{-1}\right]}^{-1}. \end{array}$$

We can obtain spectral functions $A_{L}$ and $A_{R}$ proportional to local state densities $A_{L}/2\pi$, and $A_{R}/2\pi$ in left and right reservoirs, respectively:

(A22a) $$\begin{array}{cc} A_{L} & =G_{D}\Gamma_{L}G_{D}^{†}, \end{array}$$

(A22b) $$\begin{array}{cc} A_{R} & =G_{D}\Gamma_{R}G_{D}^{†}. \end{array}$$

We can obtain functions $\Gamma_{L}$ and $\Gamma_{R}$ by the following equations, which are matrices representing the phonon lifetime in the left and right reservoirs, respectively:

(A23a) $$\begin{array}{cc} \Gamma_{L} & =i\left(\Sigma_{L}-\Sigma_{L}^{†}\right), \end{array}$$

(A23b) $$\begin{array}{cc} \Gamma_{R} & =i\left(\Sigma_{R}-\Sigma_{R}^{†}\right). \end{array}$$

We can then formulate the transmission matrix *T* of phonon waves traveling from the left to the right heat reservoirs via the interfaces of the water as follows [1,23,31]:

(A24) $$T=\Gamma_{L}G_{D}\Gamma_{R}G_{D}^{†}.$$

We can thus express the heat flow in water *J* by the following equation by integrating the product of the transmission function and the Bose-Einstein distribution [32]:

(A25) $$J=\int_{0}^{\infty }d\omega \frac{\hbar \omega }{2\pi }\sum_{n=1}^{2}\sum_{j=1}^{N}{\left\{T\right\}}_{j,n;j,n}\left[N_{0}\left(\omega \right)-N_{N+1}\left(\omega \right)\right],$$

where $\sum_{n=1}^{2}\sum_{j=1}^{N}{\left\{T\right\}}_{j,n;j,n}$ is the transmission function expressed by the trace of *T*; $N_{0}\left(\omega \right)$ and $N_{N+1}\left(\omega \right)$ are Bose-Einstein distribution functions at *j* = 0 and N+1 expressed as follows [37]:

(A26) $$N_{j}\left(\omega \right)={\left\{\exp\left(\hbar \omega /k_{B}\theta_{j}\right)-1\right\}}^{-1}.$$

Thermal conductance in water σ can then be expressed as

(A27) $$\sigma =\frac{J}{\theta_{0}-\theta_{N+1}}.$$
